# Reflections on the CODES trial for adults with dissociative seizures: what we found and considerations for future studies

**DOI:** 10.1136/bmjno-2024-000659

**Published:** 2024-06-05

**Authors:** Laura H Goldstein, Jon Stone, Markus Reuber, Sabine Landau, Emily J Robinson, Alan Carson, Nick Medford, Trudie Chalder

**Affiliations:** 1 Department of Psychology, Institute of Psychiatry, Psychology and Neuroscience, King's College London, London, UK; 2 Department of Clinical Neuroscience, Centre for Clinical Brain Sciences, The University of Edinburgh, Edinburgh, UK; 3 Academic Neurology Unit, Royal Hallamshire Hospital, The University of Sheffield, Sheffield, UK; 4 Department of Biostatistics and Health Informatics, Institute of Psychiatry, Psychology and Neuroscience, King's College London, London, UK; 5 School of Population Health and Environmental Sciences, King's College London, London, UK; 6 Research Data and Statistics Unit, Royal Marsden Clinical Trials Unit, The Royal Marsden NHS Foundation Trust, Surrey, UK; 7 South London and Maudsley NHS Foundation Trust, London, UK; 8 Department of Psychological Medicine, Institute of Psychiatry, Psychology and Neuroscience, King's College London, London, UK

**Keywords:** RANDOMISED TRIALS

## Abstract

The COgnitive behavioural therapy versus standardised medical care for adults with Dissociative non-Epileptic Seizures multicentre randomised controlled trial is the largest, fully-powered study to test the clinical and cost-effectiveness of a psychotherapeutic intervention in this population. We also explored predictors or moderators of outcomes and investigated mechanisms of change in therapy. In this current review of findings, we discuss issues related to the design of the trial and consider the study’s nested qualitative studies which were undertaken not only to shed light on the original research questions but to provide insights and recommendations for other researchers in the field of functional neurological disorder. Finally, we consider issues relating to the possible clinical application of our study findings.

## Introduction

The COgnitive behavioural therapy versus standardised medical care for adults with Dissociative non-Epileptic Seizures (CODES) multicentre randomised controlled trial was designed to test the clinical and cost-effectiveness of specifically adapted cognitive behavioural therapy (CBT) for adults with the most common disabling type of functional neurological disorder (FND), dissociative seizures (DS) which are also referred to as functional seizures and psychogenic non-epileptic seizures among other terms.^1^ DS are paroxysmal episodes of altered self-control commonly mistaken for epileptic seizures or syncope and often associated with psychiatric comorbidity and low quality of life.

Although psychotherapy has been regarded as the treatment of choice for DS, evidence of treatment effectiveness has been limited.^2^ Our programme of work has its origins in a single case study^3^ using a fear-avoidance-based CBT intervention coupled with seizure control techniques (which we refer to here as DS-CBT) with good outcomes. This was followed by an open-label study of 12 sessions of DS-CBT^4^ and then a pilot randomised controlled trial (RCT)^5^ which compared DS-CBT+standard medical care (SMC) (provided by neuropsychiatrists) with SMC alone, finding that DS-CBT+SMC was superior to SMC in reducing DS at the end of treatment. In the pilot RCT,^5^ 6 months after treatment there was an observed post-randomisation difference in favour of the DS-CBT group, but it could not be shown to be statistically significant.

**Figure 1:**
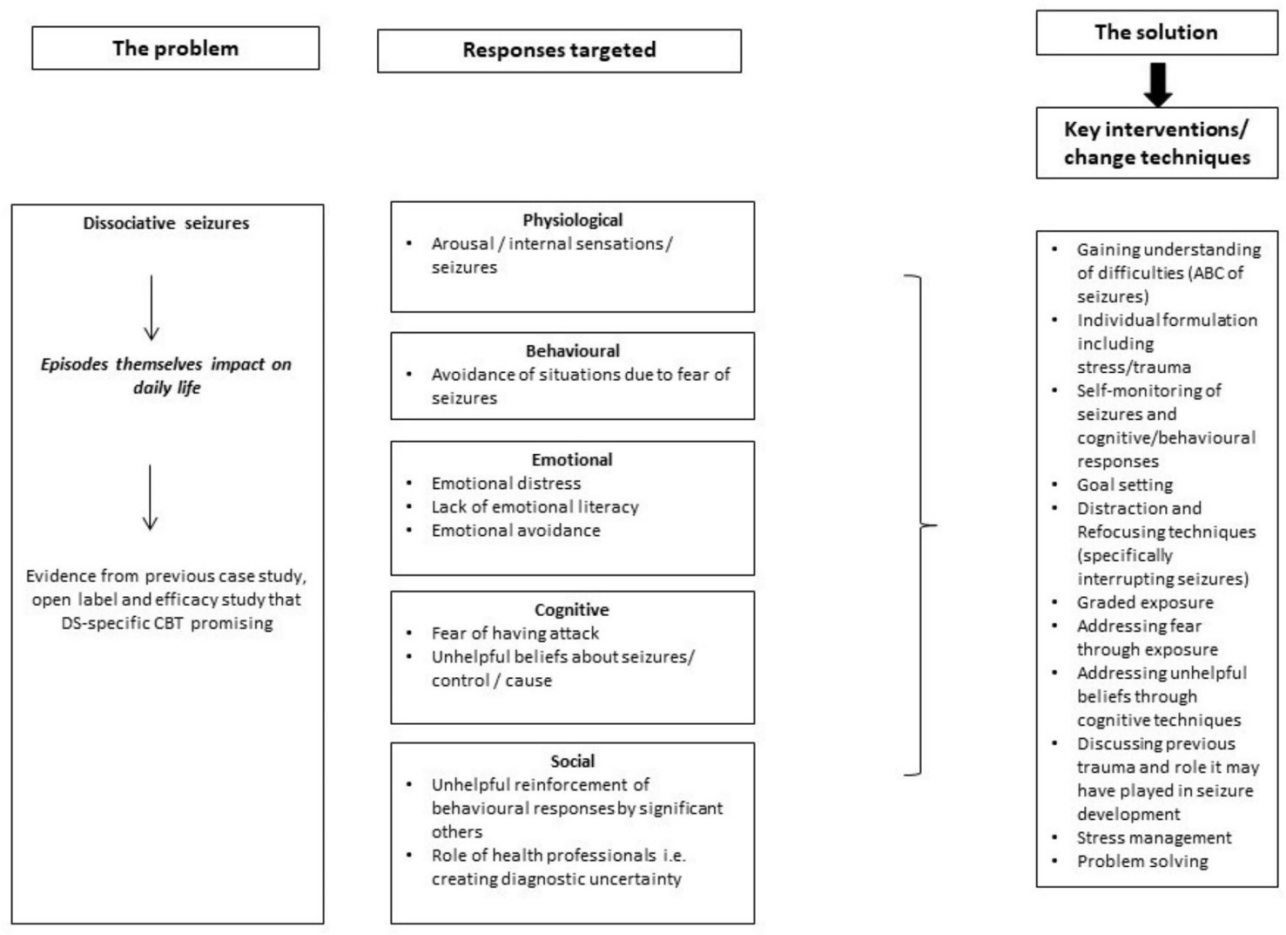
Model showing aspects of dissociative seizures and the mechanisms used to target them within our dissociative seizure-specific cognitive behavioural therapy intervention. Copyright 2021 Goldstein *et al*.^10^ This figure is licensed under the Creative Commons Attribution CC BY 4.0 licence. To view a copy of this licence visit https://creativecommons.org/licenses/by/4.0/. Figure 1 includes changes to the formatting of the original figure. ABC, antecedents, behaviour, consequences; CBT, cognitive behavioural therapy; DS, dissociative seizures.

In our model for DS-specific CBT, seizures are viewed as dissociative responses to arousal,^6^ accompanied by somatic symptoms of anxiety/panic but without subjective feelings of anxiety or panic (termed ‘panic without panic’^7^). In this model, DS are maintained by a vicious circle of behavioural, cognitive, affective, physiological and social factors of which fear and avoidance are particularly salient. Our approach incorporates Mowrer’s two-factor model,^8^ within which certain activities/behaviours or experiences are modified or avoided through fear of having seizures and the person’s lifestyle becomes increasingly restricted. This conceptualisation lends itself to the application of CBT interventions,^9 10^ particularly graded exposure to feared (avoided) situations and seizure interruption and control techniques. The components of our intervention are displayed in figure 1.

## What we did and what we found

Given the preliminary evidence that DS-CBT was helpful for DS, we were keen to ascertain whether potential treatment effects were generalisable, through a larger multicentre study where therapists varied in their knowledge and experience of DS.

**Figure 2:**
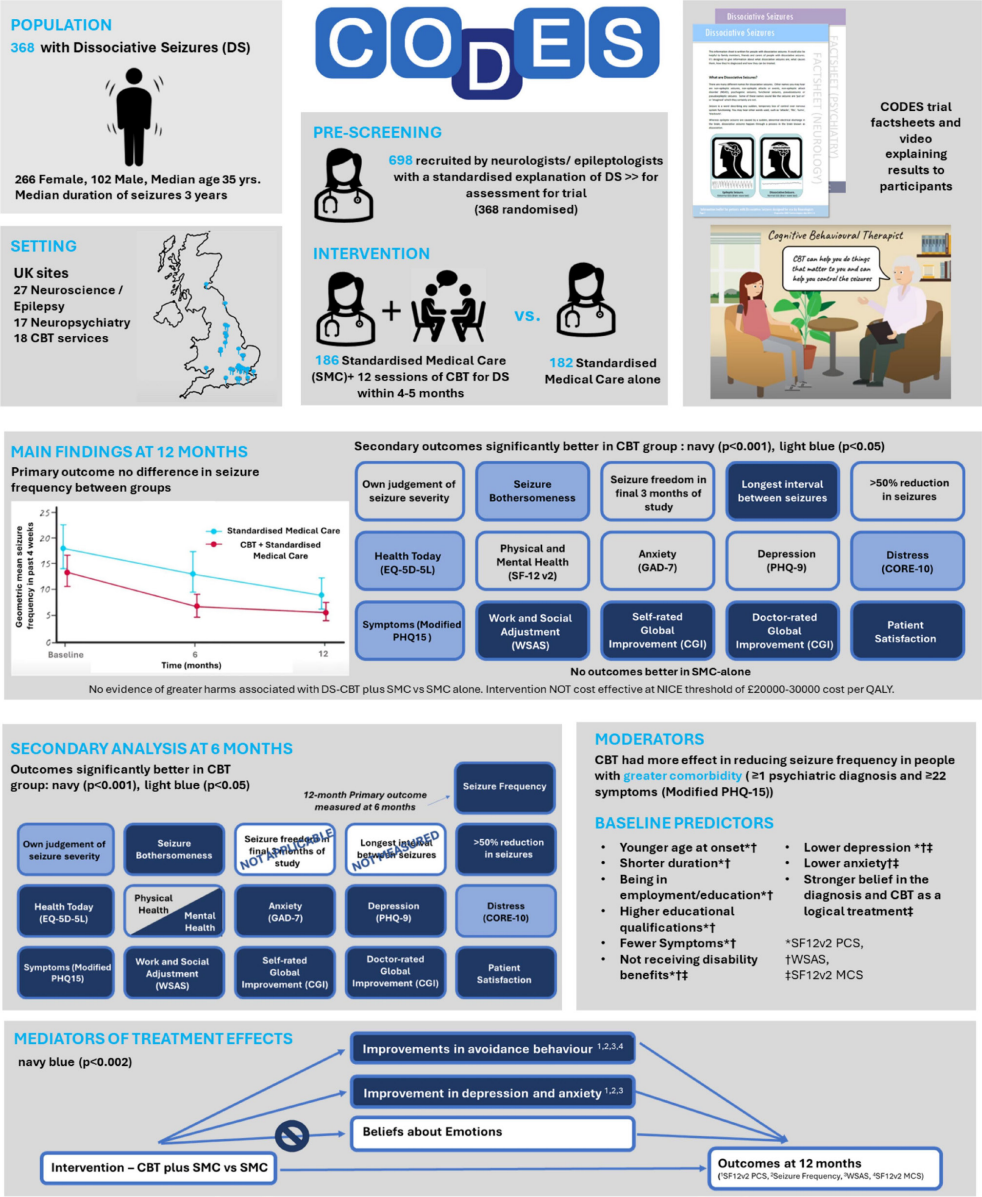
Summary of key data from the CODES trial. This figure includes still images taken from an animated video created by Science Animated (sciani.com). The originals are available at www.codestrial.org and https://www.youtube.com/watch?v=pUFKbYH7BcQ. CBT, cognitive behavioural therapy; CGI, Clinical Global Impression of Change; CODES, COgnitive behavioural therapy versus standardised medical care for adults with Dissociative non-Epileptic Seizures; CORE-10, Clinical Outcomes in Routine Evaluation 10; EQ-5D-5L, European Quality of Life 5 Dimensions 5 Levels; GAD-7, 7-item Generalised Anxiety Disorder Assessment; NICE, National Institute for Health and Care Excellence; PHQ, Patient Health Questionnaire; QALY, quality adjusted life year; SF-12v2 MCS, 12-item Short Form Survey version 2 Mental Component Summary score; SF-12v2 PCS, 12-item Short Form Survey version 2 Physical Component Summary score; SMC, standardised medical care; WSAS, Work and Social Adjustment Scale.

Here we provide a synthesis of findings from the main trial and secondary analyses^10–14^ and put the trial in context, highlighting factors that might be relevant for future studies. There is a summary of the key findings from our studies in figure 2.

The trial compared two treatment arms, DS-CBT plus SMC (n=186) versus SMC alone (n=182). Randomised patients were initially recruited from neurology/specialist epilepsy services in the National Health Service where treatment is free at the point of receipt. Importantly, rather than the equivalent of routine or standard care, SMC, as delivered in CODES, was ‘standardised and specialist’ care^10 11^ because we provided clinicians with guidelines regarding diagnosis and management (see online supplemental information). We also produced booklets specifically written for patients (see https://www.codestrial.org/INFORMATIONBOOKLETS). In addition to delivering the diagnosis in a recommended manner, neurologists and liaison/neuropsychiatrists saw patients for follow-up appointments to offer support and to prescribe pharmacotherapy when appropriate. Psychiatrists were asked not to include CBT techniques in their management.

10.1136/bmjno-2024-000659.supp1 Supplementary data

DS-CBT, delivered by therapists (who had study-specific CBT training) was manualised (materials available from codestrial@kcl.ac.uk) but, importantly, allowed flexibility for the intervention to be formulation-based, that is, tailored to the individual. Therapist adherence to the treatment approach and therapeutic alliance were measured.

### Main trial findings

Our prespecified primary outcome^9 15^ (monthly DS frequency at 12 months post randomisation) did not differ significantly between the two treatment arms, despite a 22% advantage in DS reduction in the DS-CBT+SMC group.^10 11^

There was a statistically significant advantage in the DS-CBT+SMC group in 9 of our 16 prespecified secondary outcomes, namely: more consecutive DS-free days in the past 6 months; less impact of DS on work, social and leisure activities as measured by the Work and Social Adjustment Scale (WSAS)^16^; better self-rated and clinician-rated improvement; greater satisfaction with treatment; finding DS less bothersome^17^; less distress rated on the Clinical Outcomes in Routine Evaluation-10 (CORE-10)^18^; lower symptom burden (modified Patient Health Questionnaire-15; PHQ-15)^19^ and better health rating on the European Quality of Life 5 Dimensions 5 Level (EQ-5D-5L) Visual Analogue Scale.^20^ See figure 2. No findings suggested better outcomes in the SMC-alone group. There was no evidence that any adverse or serious adverse events were related to the intervention.

The intervention was not found to be cost-effective considering the £20 000 to £30 000 cost per quality adjusted life years threshold recommended by the National Institute for Health and Care Excellence.^10^ The findings for cost-effectiveness were marginally better when estimated using the utility score (Short-Form 6-Dimension; SF-6D) derived from the 12-item Short Form Survey version 2 (SF-12v2)^21^ than from the more typically used EQ-5D-5L. It is possible however that our negative findings with respect to cost-effectiveness reflect the high levels of comorbidity^10 22^ and the very wide range of service use in our sample. In addition, we are not sure whether the EQ-5D-5L is a sufficiently sensitive measure for use in people with DS. Furthermore, as previously indicated,^10^ the time span may have been too limited to fully assess cost-effectiveness. Potentially, the effects of CBT may continue in the longer-term. We were limited to a 1-year follow-up for the trial and we did not undertake health economic modelling work to estimate the effects beyond this period.

### Secondary analyses

#### Six-month outcomes

Our prespecified^9 15^ 12-month follow-up evaluation did not include a formal analysis of outcomes at the end of treatment which broadly coincided with the 6-month post-randomisation measurement point. However, as the clarification of end-of-treatment benefits might be helpful for planning service provision, we conducted an additional secondary analysis which formally compared the groups at 6 months post randomisation. We found statistically significant differences for 13 outcomes, all in the same direction of benefit, that is, that DS-CBT+SMC was superior to SMC (figure 2). This included monthly DS frequency, that is, lower DS frequency in the DS-CBT+SMC group. Outcomes showing greater benefit in the DS-CBT+SMC group at 6 months that had not been significantly different at 12 months were self-rated seizure severity, >50% reduction in seizure frequency relative to baseline, the Mental Component Summary Score from the SF-12v2, anxiety^23^ and depression.^24^ All variables that had shown superiority in the intervention arm at 12 months showed a similar pattern at 6 months, but with larger effect sizes.

Visual and tabulated representations of the data^10–12^ suggested that rather than the benefit from DS-CBT+SMC diminishing between 6 and 12 months post randomisation, the more modest between-group results at 12 months largely resulted from improvements in the SMC-alone group across the follow-up period, accounting for the apparently smaller effect sizes at 12-month than at 6-month follow-ups. We speculate that this derived from support provided during SMC sessions which were spread across the 12-month follow-up period, and potential exposure to self-help websites and thereby seizure control strategies in the SMC group. Although both groups were exposed to SMC which included a discussion, but not the practice of distraction techniques, the DS-CBT intervention directly included DS-control strategies and may therefore have led to faster adoption of such techniques by participants than might have occurred in the SMC-alone group. In the absence of a completely inactive control group, we could not be sure, however, that this pattern of change in the SMC-alone group did not simply represent the natural history of DS and/or regression to the mean. Overall, inspection of our data does not support others’ suggestions that our treatment did not sustainably reduce DS frequency.^25^ It is possible though that extending therapy time beyond 12 sessions may have been additionally beneficial in continuing to assist participants practice and apply the DS-control techniques, as well as other therapeutic strategies, particularly given the complexity of the participants.^10 22^

This secondary analysis showed that it is important to undertake both short and longer-term follow-ups in treatment trials. Short-term outcome evaluations alone (eg,^26^) may give a distorted impression of the benefit or otherwise of the target intervention. It has been suggested^27^ that our cost-effectiveness analysis might have yielded better results if conducted at 6 months rather than 12 months post randomisation, since seizure frequency differed between groups at 6 months, but we did not investigate this possibility.

#### Treatment moderation

One issue that we did not address in our main outcome accounts^10 11^ was who might particularly benefit from DS-CBT, or what might predict treatment outcome. We explored this through a further secondary analysis to examine moderators of treatment effects, that is, which patient baseline characteristics might interact with CBT to influence outcomes. Putative moderators are baseline characteristics thought to be associated with more improvement in the DS-CBT+SMC arm.^13^ Where no moderators were found, baseline predictors of outcome across both interventions were investigated.^13^ Here we chose four outcomes, all at 12 months post randomisation: monthly DS frequency (our main primary outcome); the functional impact of DS as measured by the WSAS; and two quality of life measures, the Physical Component Summary score (PCS) and Mental Component Summary score (MCS), both from the SF-12v2. We prespecified putative moderators based on the literature.

Importantly, we found that DS-CBT was more effective than SMC-alone in reducing DS frequency for participants with at least one comorbid diagnosis on the Mini-International Neuropsychiatric Interview (M.I.N.I.)^28^ or having a score of at least 22/30 on the modified PHQ-15 (figure 2). For those with no comorbid M.I.N.I diagnoses or fewer symptoms on the modified PHQ-15, DS-CBT did not lead to a significantly greater reduction in DS frequency than SMC-alone. Our fear-avoidance-based DS-CBT approach might have specifically benefitted more complex patients because key intervention change techniques are likely to focus on aspects of comorbidity.^13^ Thus, for example, given the relatively high prevalence of agoraphobia (45%), major depressive disorder (31%), generalised anxiety disorder (29%), post-traumatic stress disorder (23%) and social anxiety disorder (20%) in our sample at baseline^10 22^ our focus on dealing with avoidance behaviour, anxiety, depression and to a more limited extent trauma may have been most useful for participants reporting these areas of difficulty.

Several baseline characteristics predicted outcome as measured by the WSAS, SF-12v2 PCS and SF-12v2 MCS across both intervention arms. Of note, predictors with larger effect sizes tended to be those related to socioeconomic characteristics such as being employed or in education, not being in receipt of state financial benefits and higher educational attainment. Some DS-related characteristics such as age at onset or duration of DS disorder, and anxiety and depression, were also predictive of outcome across both intervention arms. DS semiology did not predict outcome. Strength of belief in diagnosis was predictive of improvement across both arms on SF-12v2 MCS scores.

The suggestion that more complex patients might particularly benefit from CBT in terms of DS reduction when the comparison was SMC-alone raises the possibility that SMC, which was effectively enhanced treatment as usual, might be appropriate for less complex patients. However, services contemplating only offering DS-CBT to more complex patients would have to be able to deliver SMC to less complex patients, using the same pathway as evaluated in the CODES trial.

It has been speculated that more positive trial effects might not have been found due to high rates of maladaptive personality markers in participants (that is, scores on the Standardised Assessment of Personality Abbreviated Scale, Self-Report scale; SAPAS-SR^29^) and whether patients with this profile might have fared better with a more psychodynamically-oriented intervention.^2^ However, in our secondary analysis, our measure of maladaptive personality traits (SAPAS-SR) was found neither to moderate the treatment effect nor to predict treatment outcome. We acknowledge that a clinical assessment of personality disorder might have been more informative but this was outside the scope of the assessments we could undertake within the study. The heavy measurement burden for participants also meant that we did not include measures of dissociation or specific measures of trauma history that might also have helped clarify the patients for whom DS-CBT was particularly effective; this should be considered in future studies.

#### Mediation of treatment effects

Our final secondary analysis evaluated putative treatment mechanisms by examining putative mediators of the effect of DS-CBT+SMC on outcomes^14^ finding that DS-CBT effects on 12-month seizure frequency, functional impact and quality of life measures were mediated by changes in avoidance, anxiety and depression at 6 months (figure 2). DS-CBT did not bring about an improvement in another putative mediator, beliefs about emotions (as measured by the Beliefs about Emotions Scale; BES),^30^ although improvements in BES scores were associated with improvement in some outcomes. Estimates of mediated (indirect) effects tended to be small, with the largest mediated effects for avoidance and the WSAS.

The finding that avoidance behaviour mediated change in several outcomes supported our theoretical fear-avoidance model on which DS-CBT was partly based,^3 8 9^ and indicated that therapists were for, the most part, addressing avoidance behaviour during therapy. However, our analyses indicated that the mediated effects were relatively small; perhaps targeting beliefs about emotions and emotional avoidance more directly may be helpful in influencing outcomes. Of course, unmeasured mediators related to the therapeutic techniques used and to the therapeutic relationship may also have revealed more about the mechanisms of the intervention.

### Choice of outcome measures

We chose monthly DS frequency as our trial primary outcome partly because the funder had identified seizure frequency as an important outcome (https://njl-admin.nihr.ac.uk/document/download/2023204) and partly because we had collected previous data on this measure,^5^ enabling data-based power analysis. It is also the symptom with which patients present, and for which they seek diagnostic evaluation. It is worth noting, however, that recording seizure diary data over a protracted period is challenging for patients. It can lead to variable amounts of missing data and measurement error, which requires careful consideration of how the outcome should be constructed from the weekly counts available.^10 11^ Moreover, DS frequency was a highly skewed outcome. Although we strictly complied with our analysis plan and were fully transparent about all statistical methods applied, including imputation steps, we therefore acknowledge that other solutions were available. Similar issues with recording seizure frequency in epilepsy have also been highlighted, suggesting it is not an optimal primary endpoint in a heterogeneous population. Other options might have been to adopt seizure freedom or the longest number of consecutive DS-free days as primary outcomes but we opted to include these as secondary outcomes, with data on these measures being reported in our main publications.^10 11^

Others^31^ have questioned whether another measure such as productivity might be more informative, and a recent meta-analysis has suggested that non-seizure outcomes are responsive to psychological treatment in patients with DS.^32^ Given that CODES secondary outcomes showed between-group differences in 9 of 16 secondary outcome measures^10 11^ it has also been questioned whether our primary outcome measure at 12 months post randomisation best represented the effectiveness of DS-CBT.^33^ Discussion of outcome measurement in FND more widely^34^ has advocated the use of core and supplementary measures. Several of these were incorporated in our selection, including the core symptom outcome measure recently recommended,^34^ that is, patient and clinician-rated global impression of improvement, based on the Clinical Global Impression of Change (CGI) measure.^35^ We found a between-groups difference in how bothersome^17^ participants found their seizures, even though there was no difference in seizure severity at 12 months post randomisation. This lends support to the future adoption of a self-rated CGI, or ‘seizure bothersomeness’/ ‘seizure severity’ rating, which is arguably a more specific form of self-rated seizure outcome, as a primary outcome.

Our qualitative work with trial participants^36^ supports the suggestion that changes in perceptions of the debilitating effects of DS may be much more important than the exact number of seizures participants have. A seizure in the middle of a busy workplace leading to an ambulance being called is very different from one occurring in bed just before sleep when the individual has found they can delay seizures using seizure control techniques. Therapists also acknowledged the value of patients being able to stall seizures to permit engagement with important activities.^37^

Finally, therapists beyond our study have expressed uncertainty over whether they can or should target DS symptoms directly rather than addressing quality of life more broadly.^38^ The CODES trial showed that specific seizure control techniques can be incorporated into a psychological therapy, analogous to panic control techniques. So even if a trial (or a clinical service) focuses on quality of life as a primary outcome, measuring seizure occurrence or impact in some manner appears relevant.

### Choice of comparator group

The comparator condition in a trial can have a dramatic effect on the apparent effectiveness of the intervention. Participants in the SMC arm of the CODES trial did not receive ‘treatment as usual’ which in many parts of the UK and further afield would often mean ‘no treatment’ or attempted treatment from a mental health professional without experience of DS.

All participants experienced a neurologist/epilepsy specialist, trained in DS communication, explaining and providing well-presented written material about the diagnosis. Participants’ mean ‘agreement with the diagnosis’ after this process was 8 on Likert scale of 0–10 where 10 was complete agreement.^10^ So even before randomisation, most participants were perhaps more ready for treatment and understood the role of psychological therapy than a patient not having been given a clear diagnosis or written material. This may, therefore, have better prepared those patients subsequently receiving DS-CBT. However, the wider benefit of such an approach has been shown by a small study in Australia which highlighted how providing even these basic components to individuals with DS can be associated with much lower readmission rates and health economic costs.^39^

The standardised treatment in CODES consisted of seeing a liaison psychiatrist or neuropsychiatrist, experienced in DS, for up to a year. Although psychiatrists were asked not to include elements of the intervention, this is not a typical ‘treatment as usual’ scenario, and the comparator treatment is arguably one that could itself have been the focus of an intervention study. The fact that the DS-CBT plus SMC arm showed differences in so many clinically relevant secondary outcome measures in this context is arguably much more impressive than if the comparator arm had been ‘treatment as usual’. However, it is also necessary to acknowledge that our positive outcomes might in some, unspecified way, reflect synergy between DS-CBT and SMC and we cannot determine whether DS-CBT might have been less effective without adjunctive SMC. Future studies may wish to examine in greater detail the effectiveness of our model of SMC as compared with either a waiting list control or treatment as usual. The effect of spacing/frequency of SMC sessions could also shed light on its effectiveness.

Could the comparison group have been given a different psychological intervention? Even more psychodynamically-oriented treatments^40^ may contain some CBT-based elements targeting seizure control. It seems likely, based on other data, that the sample size needed to demonstrate a difference between two active psychotherapies would be so large as to not be feasible for this kind of design. However, a more inactive comparison such as progressive muscular relaxation could control for therapist time and attention and may be worth considering.

### Context for implementation

In the CODES trial, we created a clinical pathway that included neurologists/epilepsy specialists, liaison or neuropsychiatrists and CBT therapists. This pathway was embedded in a UK health service with variable service provision for patients with DS and limited opportunities for neurologists to refer patients for psychotherapy.^41^ Funding regulations meant we could not directly pay clinicians for their time in the study with research funds, so we had to establish clinical pathways relying on the enthusiasm of interested services and clinicians. It is likely that SMC as provided in CODES was shaped by this enthusiasm. We did not specifically explore the moderating effect of site on outcomes but the effects of the randomisation stratifier 'site' were allowed for in all our analyses.^10–14^ Future studies might wish to consider systematically auditing the content of SMC for fidelity, rather than simply providing guidance to clinicians as to its content in the manner we employed here.

People attending their first psychiatric appointment were no different from those who did not, in terms of most baseline characteristics, including confidence in the diagnosis,^42^ suggesting that neurologists should be agnostic about who they refer for further treatment. Psychiatrists, who assessed patients 3 months after the neurologist, felt that patients they saw in the trial were generally more accepting of their diagnosis than other patients they saw, possibly as a result of the way neurologists had explained the diagnosis as well as the support materials made available to the neurologists and the patients.^43^ Therapists generally considered that those patients receiving DS-CBT in the trial seemed to present with a better understanding of their diagnosis than those seen in other contexts.^10 37^

We acknowledge that the CODES pathway infrastructure has not been maintained across all participating services although, following study completion, a survey of participating neurologists showed the majority were keen for the pathway to continue.^41^ Those therapists wanting to implement our DS-CBT approach should attempt to do so in association with input from neurologists and psychiatrists with expertise in this disorder. As noted previously the multidisciplinary CODES pathway^10^ may limit the extent to which patients feel ‘abandoned’ by medical staff following diagnosis and may prepare patients better for psychotherapy, as well as facilitate more cohesive interdisciplinary working.^10^ A consistent message from the multidisciplinary team is likely to be more powerful.

Our assessment of treatment fidelity^10^ involved the independent rating of pseudorandomly-selected recorded DS-CBT sessions, conducted by our therapists who had undergone study-specific training. The therapists were also allocated to supervision groups led by therapists experienced in delivering our treatment model. Our ratings suggested that DS-CBT+SMC can be implemented in a range of services. However, adhering to a DS-CBT treatment protocol, even if it is formulation-based and tailored to the individual, may be challenging for therapists who would otherwise adopt a more eclectic psychotherapeutic approach.^37^

A further consideration regarding implementation, especially in less specialised services is the provision of education about FND in general and DS in particular for staff. Some trial psychiatrists^43^ and therapists^37^ commented on the complexity of the patient group and the potential for damage to a patient’s progress by incorrect references to the patient possibly having epilepsy or needing anti-seizure medications.^43^ Both groups suggested that more experienced staff would be best placed to treat patients with DS. This means that services wishing to implement CODES-informed treatment pathways will not only need to consider the expertise of those delivering therapy but also of those providing supervision.

We also acknowledge the lack of diversity in our participant sample, with approximately 90% of our randomised patients self-identifying as white. While this may have derived from our eligibility criteria, whereby we excluded people who would not have been sufficiently fluent in English to complete measures and therapy without an interpreter, we cannot be sure about the extent to which our findings would apply to a broader demographic and the cultural competencies of therapists would also require consideration. Similarly, we cannot conclude that our findings would be generalisable to individuals with an intellectual disability or concurrent active epilepsy since such people were not included in the study. Nonetheless, as we have indicated elsewhere^22^ in addition to a typical proportion of women to men for a DS sample, our sample was broadly generalisable in terms of duration of symptoms, mean number and profile of comorbid psychiatric diagnoses (although people with the most severe psychopathology requiring crisis intervention were excluded from randomisation), at least moderately severe functional impairment and appreciable levels of avoidance behaviour and a range of symptoms on the modified PHQ-15 that were generally similar to those documented for other neurology outpatients with symptoms not explained by disease.

## Conclusions and lessons learnt from the trial

CODES was a clinical trial, developed over a succession of studies^3–5^ in a condition that remains only partially understood, so there is much to reflect on with respect to the nature of DS itself, the way that we measure it and the best ways to test potential treatments.

It is interesting that the primary outcome of seizure frequency was negative at 12 months. Simple frequency of seizures may not be as important to people with DS as their impact or ‘bothersomeness’. Given the importance of the primary outcome in determining treatment effectiveness, researchers should be very clear about their choice. Considerations in this context include the timing as well as types of measure. Timings depend on whether one wants to demonstrate immediate or long-term benefits. Long-term outcome is difficult, for example, due to waning of treatment effects, possible improvement in the control arm over time, or lack of power due to loss-to follow-up. The variable itself should measure the construct of interest and be feasible. In CODES we examined seizure frequency, but the construction of this variable from diaries was not straightforward.^10 11^

The consequences of choosing a primary outcome measure should not mean that secondary outcome measures are ignored. We also found it useful to have prespecified our intention to undertake secondary explanatory analyses and would advocate this in future studies. We showed that participants with additional psychiatric comorbidity were especially likely to benefit from the treatment, indicating that complexity (reflecting psychiatric and somatic comorbidities) should encourage therapy, rather than the converse.

What advice do we have for those embarking on new trials for DS? The trial design meant that we could not pre-assess for suitability for CBT but had to randomise all those who were eligible/willing to be randomised. We could not let patients delay the onset of treatment till it might have been more convenient for them because of the fixed study timelines. Altering the time taken to start treatment might be something for future triallists to consider. New insights into the underlying mechanisms of DS may inform changes to treatment. It has been suggested^44^ that psychotherapeutic interventions specifically targeted towards particular patient characteristics and symptom profiles might be more effective than our blanket application of DS-CBT, although evidence for this is lacking. The treatment was manualised but could still be individualised and was therefore flexible to patient heterogeneity. The role of supervision was especially important. We cannot be certain whether the range of patient complexity present in our trial required a yet larger sample size to address the potentially increased variance introduced by clinical heterogeneity in the sample. For example, we have reported that while there was a median of 2 current psychiatric diagnoses on the M.I.N.I. in the sample, the range was 0–8 diagnoses.^11^ Neither do we know whether the heterogeneity of the sample meant that the study included individuals unlikely to respond to the study intervention. However, future studies may wish to consider whether to include larger samples or samples/subgroups of participants with more homogeneous profiles to consider further who might respond best to the intervention being tested.

We developed a treatment manual and patient materials that could facilitate the formulation-based delivery of DS-CBT, and which led to acceptable levels of adherence.^10^ However, while therapists working outside the requirements of our treatment protocol might want to adopt a more eclectic approach and therefore may not consider that a comprehensive treatment manual for patients with DS is feasible,^38^ it is important to note that a truly individualised and eclectic psychotherapeutic approach is both difficult to describe and replicate and, while attractive to many therapists, such an approach has not yet been subject to rigorous evaluation.

The conduct of the trial benefitted enormously from the patient and public involvement (PPI) aspects of the study. We were able to include PPI at the stages of study design, management and dissemination.^10^ Involvement in ongoing trial management was reflected in the membership of our Trial Management Group and Trial Steering Committee (two people per committee) and we provided training (from an external agency) to facilitate their early involvement in these oversight committees. Meeting agendas included standing items to encourage their contributions if they did not speak at other times in the meetings. We provided additional opportunities for them to communicate issues to the chief investigator and trial manager. We encouraged authorship on papers but also acknowledged that, for some, the persisting stigma attached to making public a mental health diagnosis rendered this undesirable. The involvement in dissemination extended valuably beyond academic publications, and included an output designed for a wider audience (https://www.youtube.com/watch?v=pUFKbYH7BcQ). While now clearly required by many funders we would strongly recommend that future studies take seriously the incorporation of PPI from the earliest stages of study conception and design and build in support mechanisms for those people providing this important aspect of study delivery. Researchers should not be oblivious to the possibility that PPI can trigger distress in those providing such input due to reminders about their own clinical histories.

Many aspects of the trial went well. The trial recruited the expected numbers at the correct time. This sends an important message for those considering developing or funding clinical trials in FND, that is, that recruitment is feasible. Indeed, our experience was that participants were nearly always happy to have their problems taken seriously by a research team, and saw the trial as offering them hope.^36^ We demonstrated the importance of having approval to obtain some demographic and clinical information on patients screened but not necessarily recruited to the trial.^10 11 42 45^ We had this information due to our two-stage recruitment approach, which proved very useful for characterising the trial sample and assessing its generalisability. The trial showed that neurologists welcomed being given guidance on how to communicate the diagnosis and have supporting written materials to give to patients.^41^ We showed that therapists without a background in DS could be trained to deliver treatment with acceptable fidelity. Perhaps most strikingly, the formation of a large national clinical network with interdisciplinary collaboration between psychology, psychiatry and neurology was, in itself, an achievement in a disorder in which care has traditionally been historically ignored and fragmented between specialties.
