# Supplementary material for: Reflections on the CODES trial for adults with dissociative seizures: what we found and considerations for future studies
Source: BMJ Neurol Open. 2024 Jun 5;6(1):e000659. doi: 10.1136/bmjno-2024-000659 (PMC11163627; doi:10.1136/bmjno-2024-000659)
Supplement: Supplementary data [file bmjno-2024-000659supp001.pdf]

Reflections on the CODES trial for adults with Dissociative Seizures: what we found and considerations for future studies

Laura H. Goldstein, Jon Stone, Markus Reuber, Sabine Landau, Emily J Robinson, Alan Carson, Nick Medford & Trudie Chalder

Supplementary Information

Links to NIHR Journals Library for the guidance materials given to neurologists and psychiatrists providing standardised medical care:

https://www.journalslibrary.nihr.ac.uk/publications/hta25430/12-26-01-supp4.docx,
https://www.journalslibrary.nihr.ac.uk/publications/hta25430/12-26-01-supp5.docx,
https://www.journalslibrary.nihr.ac.uk/publications/hta25430/12-26-01-supp6.doc,
https://www.journalslibrary.nihr.ac.uk/publications/hta25430/12-26-01-supp7.docx
https://www.journalslibrary.nihr.ac.uk/publications/hta25430/12-26-01-supp8.docx"https://www.journalslibrary.nihr.ac.uk/publications/hta25430/12-26-01-supp8.docx.
